# Content-Specific Working Memory Modulation of the Attentional Blink

**DOI:** 10.1371/journal.pone.0016696

**Published:** 2011-02-02

**Authors:** Elkan G. Akyürek, Ali Abedian-Amiri, Sonja M. Ostermeier

**Affiliations:** University of Groningen, Groningen, The Netherlands; University of Regensburg, Germany

## Abstract

Three experiments were conducted to investigate the effects of working memory content on temporal attention in a rapid serial visual presentation attentional blink paradigm. It was shown that categorical similarity between working memory content and the target stimuli pertaining to the attentional task (both digits) increased attentional blink magnitude compared to a condition in which this similarity was absent (colors and digits, respectively). This effect was only observed when the items in working memory were not presented as conjunctions of the involved categories (i.e., colored digits). This suggested that storage and retrieval from working memory was at least preferentially conjunctive in this case. It was furthermore shown that the content of working memory enhanced the identification rate of the second target, by means of repetition priming, when inter-target lag was short and the attentional blink was in effect. The results are incompatible with theories of temporal attention that assume working memory has no causal role in the attentional blink and support theories that do.

## Introduction

Attention cannot be deployed efficiently to more than one perceptual event within about 500 ms. The attentional blink phenomenon illustrates this difficulty in a steep drop in successful report of the second of two briefly presented visual target stimuli during this critical interval [1], [2]. The precise source of this temporal limit on attention is a matter of some debate (for an overview, see [3]). Several influential theories of temporal attention have attributed a crucial role to memory consolidation [4]–[7]. In these and similar accounts, basic visual feature perception can operate in parallel for multiple stimuli, but more elaborate processing, such as associated with memory consolidation, binding, (object) identification and response selection, cannot. The essence of these accounts is a division between two phases or stages of processing; an early stage that is more or less capable of processing several stimuli simultaneously without strong interference, and a subsequent late stage that is not (i.e., it is serial). In an attentional blink task, when the observer is processing the first target and consequently unable to put the second target stimulus through this late processing phase (the “second stage”), and thereby consolidate it to some degree, its partially processed features suffer from decay and interference as they linger in the early processing stage. Thus, the loss of information in the parallel stage is caused indirectly by the bottleneck in the serial processing stage, and so gives rise to the attentional blink.

This view has been challenged by models that more or less explicitly claim they do not need to suppose such ‘capacity limitations’ on the perceptual processing that pertains to the attentional blink [8], [9]. These models of the attentional blink instead attribute an important role to the inhibition of distracting stimuli on the one hand and the selection of the target stimuli on the other. In the model by Di Lollo and colleagues [8], the attentional set necessary to select the target stimuli is thought to require continuous maintenance from an executive control function. According to this model, the control function is also needed to process the target stimuli, so that the attentional set becomes vulnerable when the control function is needed to process the first target. The arrival of a distractor stimulus then triggers a switch of the attentional set, thus causing the blink. Somewhat similar mechanisms are modeled by Olivers & Meeter [9], in that the target stimulus is thought to elicit a transient ‘boost’ of attention, and the subsequent distractor elicits an opposite, inhibitory response. Because the boost mechanism is somewhat slow, it ends up boosting the distractor stimulus that follows on the first target. This in turn requires a stronger-than-usual inhibition response, which is thought to constitute the attentional blink.

Arguments in favor of both ‘camps’ have been brought forward. Against strict capacity limitations, based on the number of individual stimuli, speak results related to Lag 1 sparing, which is the absence of the blink if the two targets follow each other immediately, without intervening distractors. It has been shown that Lag 1 sparing can be extended in time, if observers are asked to report an uninterrupted sequence of targets; no attentional blink is evident from such sequences [8], [10]. However, capacity-based models could explain the report of multiple sequential targets by referring to chunking mechanisms that would allow multiple stimuli to be part of one larger representation in memory. Against distractor-based accounts speak studies that showed residual AB effects on sequential targets when data showing extended sparing were analyzed with a strict method to control for multiple target contingencies [11], [ see also 12], and studies showing that the attentional blink can be elicited without using distractors between targets [13], [14]. Still, one could suppose that a distractor is not strictly necessary if the decay of the attentional set over time was fast enough, or if the absence of a distractor could also be sufficient to trigger an inhibitory response under certain conditions. Thus, the debate has not been settled conclusively (for a hybrid state-of-the-art model, see [15]).

A fairly direct test of the assumptions of these two types of models is to examine the impact of working memory load on attentional deployment. Models assuming that memory consolidation is (part of) a limited, serial process, which underlies the blink, would predict that increased load on memory should modulate blink magnitude. Models that do without this assumption of limited processing would not predict such modulation. Several studies on the interaction between working memory and temporal attention have been reported. Some showed that working memory load increased overall task difficulty, but showed no modulation of blink magnitude as determined by a relative increase of difficulty at short temporal lag between targets, compared to long lag [16], [17]. Likewise, in a test of individual differences, measures of working memory capacity failed to correlate with blink magnitude [18]. However, other studies did show blink-specific modulation of attention by memory load in behavioral and electrophysiological paradigms [19], [20] (for memory encoding difficulty see also [21]), as well as in correlations between individual operational working memory span and the blink [22], [23]. The reason for the discrepant findings seems to lie in the processing mode of working memory required at the time of attentional deployment. In the studies that showed no relation between working memory and the blink, observers were asked only to maintain items in memory for recall later, or were tested on memory capacity mostly. In the positive studies, observers had to access memory when the first target appeared (to perform a matching task), or were tested on operational span more explicitly. This suggested that active use or access of memory is required for the interaction between memory and temporal attention to appear.

From this literature it can be concluded that these studies do in principle support (operational) memory-based models of the attentional blink, but also leave some questions unanswered. First, the possibility remains open that it is not working memory involvement per se that is the critical factor, but an executive function that might also be involved in accessing or updating working memory. In the model by Di Lollo and colleagues [8], a function of this kind is deemed necessary to process the targets and to maintain the attentional set. Second, in the Akyürek, Hommel, and Jolicœur [19] study, the need to match the identity of the first target to the contents of working memory may be construed as potentially involving a switch in attentional set or filter (sometimes also referred to as a “gate”). Such a reset of the attentional filter could potentially have a blink effect in the model by Olivers and Meeter [9]. The same confusion between attentional switching ability and memory operations may be supposed to underlie individual ability tests as well. One might thus again assume that it is not working memory involvement that is at the core of the reported interference, but rather this demand on the attentional filtering function. This account does necessitate the additional assumption that a larger memory set translates into a prolonged involvement of the attentional gate, and thereby leads to an increased attentional blink.

The purpose of the present study was to take away the potential source confusion between working memory and executive or attentional gating functions, and thereby to settle the issue of working memory involvement as a fundamental factor in the attentional blink. This was realized by implementing two working memory conditions that each should involve executive and gating functions in a similar way (i.e., increasing involvement of the executive or prolonged gate switching), but should be different from a memory perspective. In particular, one condition featured categorical similarity between the items in working memory and those relevant to the attentional part of the task (alphanumeric characters), whereas the other did not (colors). In terms of the working memory model proposed by Baddeley [24], the color and alphanumeric information could be held in different subsystems (the visuospatial sketchpad and the phonological loop, respectively). Therefore, increased interference between the alphanumeric attentional task and the alphanumeric working memory task was expected in the form of an increased attentional blink, compared to the condition that featured a color memory task.

## Methods

### Experiment 1

The aim of Experiment 1 was to establish whether the type of content in working memory has an effect on the efficiency of attentional deployment. To this end, two types of working memory content were used in a memory-attention dual task similar to the one used by Akyürek, Hommel, and Jolicœur [19]. In this task, observers are asked to match T1 to a previously presented memory set, and it was shown that such access to memory interferes with the processing of T2. The first type of working memory content used presently was different from the type of items used in the attentional task; participants were asked to remember a number of colors and to attend to two alphanumeric targets in a rapid serial visual presentation (RSVP). The second type of content was similar to that used in the attentional task (without repeating the actual items); participants were asked to remember alphanumeric stimuli and attend to (other) alphanumeric stimuli in the RSVP. The prediction was that if the type of content (that is actively accessed) in memory matters to attentional deployment, a difference between these two conditions should be observable on the identification performance on the second target.

#### Participants

Thirty-four students of psychology (23 female, 11 male) at the University of Groningen participated in the experiment for course credit. Participants were unaware of the purpose of the experiment and reported normal or corrected-to-normal vision. Informed consent was obtained in writing and the study was approved by the Ethical Committee Psychology (ECP) of the University of Groningen before it was conducted. Mean age was 20.4 years, with a range between 18–32 years. Five participants were excluded from analysis because their performance on the second task (conditional on T1, see below) fell below the pre-specified cut-off value of 10% correct in one or more of the experimental conditions. This value was chosen to exclude participants who misunderstood the task, or for whom the task was clearly too difficult. Mean age of the remaining 29 participants (19 female, 10 male) was 20.7 years, with the same range as the full sample.

#### Apparatus and stimuli

Participants were seated individually in a dimly lit testing cabin. The distance from their chair to the 22” CRT screen on which the stimuli were presented was approximately 50 cm. The screen refreshed at 100 Hz at a resolution of 800 by 600 pixels in 16 bit color. The experiment was programmed in PST E-Prime 1.2 and ran on a computer running the Microsoft Windows XP operating system. Responses were recorded with a standard keyboard. A light gray background (RGB 192, 192, 192) was maintained throughout all trials of the experiment. Stimuli consisted of letters and digits, which were drawn in bold 36 pt. Courier New font, with the exception of the fixation cross (“+”) in bold 18 pt. font. The digits were drawn randomly without replacement from 2–9, and the letters from the full alphabet. The stimuli were mostly presented in black (RGB 0, 0, 0), except for the targets and the items in the memory set. The items in the memory set each had a unique color, drawn from the standard 16-color palette, with the exclusion of black, dark gray (RGB 128, 128, 128), light gray (identical to the background color), and red (RGB 255, 0, 0). Depending on the experimental condition, the first stimulus could either match the color of one of the items from the memory set, or have another unique color from this set (i.e., in the no-match condition). The second target stimulus was always red.

#### Procedure and design

**Figure 1:**
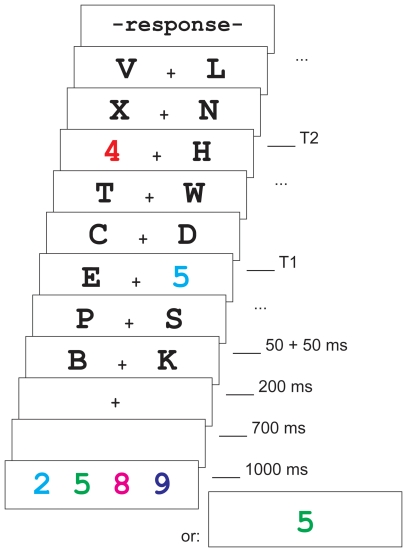
The experimental procedure as used in Experiment 1. The trial is depicted from the onset of the memory set (100 ms after trial start) until the onset of the response screens (max. 1500 ms each). One or more similar frames are indicated with dots (“…”). The colors are not meant to precisely reflect the actual RGB values used in the experiment and are provided for illustration purposes only.

The experiment had a total of 768 experimental trials divided between 4 blocks, and 48 practice trials. The experimental trials were self-paced and initiated by pressing Enter. At 100 ms after the start of the trial the memory set was shown for 1000 ms, followed by a blank screen for 700 ms. The memory set consisted of either 1 or 4 digits, depending on the experimental condition. The probability of each was 50%, and was otherwise randomly drawn. The digits in the memory set each had a unique color (specified above). Depending on the task instructions for the current block, which were shown before the start of each block, participants were asked to memorize either the colors, or the identities of the digits in the set (50% probability, presented in counter-balanced blocks). After offset of the memory set, the fixation cross appeared for 200 ms, after which two synchronized and simultaneous stimulus streams started. The streams consisted of 20 frames, each visible for 50 ms, and followed by a blank interval of 50 ms. The two target stimuli (T1 and T2) in these streams were digits, drawn from 2-9, while the remaining distractor items were letters drawn separately for each of the two streams from the full alphabet. T2 was never the same digit as T1, nor the same as any digit presented in the memory set. The streams were aligned to the center of the screen and 128 pixels apart on the horizontal axis. T1 appeared at the fifth or the seventh position in the stream, and could appear in either the left or the right stream. T2 followed T1 at a lag of 1, 3, or 8, and could appear in either the left or the right stream independently. As before, these possibilities were evenly distributed across all conditions and presented in a randomly drawn trial sequence. A 100 ms blank screen was shown after the streams had ended, after which two successive prompts asked participants to enter their responses to T1 and T2, respectively. The prompts remained on screen for 1500 ms each, or until a response was given. To indicate whether T1 was also part of the memory set, participants were instructed to press the 1 key (labeled with a sticker that depicted a triangle) if it was, and the 0 key (labeled with a square) if it was not. In the color task condition, T1 should be considered part of the memory set if it had the same color as one of the items from the set, regardless of its identity. In the identity task, the digit presented as T1 should match one of the items from the set, regardless of its color. T2 could be identified by pressing the corresponding number between 2 and 9 on the keyboard. The experimental design is illustrated in Figure 1.

Accuracy was analyzed in a repeated measures analysis of variance with three variables. The first variable was T1-T2 Lag (levels 1, 3, and 8), the second variable was Load (1 or 4 items), and the third variable was Task (color or identity). T2 accuracy was computed as the percentage of correctly identified stimuli, given that the response to T1 was correct (T2|T1). In case of significant tests of sphericity, the degrees of freedom were adjusted using the Greenhouse-Geisser epsilon correction.

### Experiment 2

Experiment 2 was designed to isolate the task-relevant feature of the stimuli that were part of the memory set. Thus, when the task for the observers was to memorize colors, the items in the memory set were small colored discs. When the task was to memorize (alphanumeric) identity, the items were changed to digits. If the null effect on T2 performance for the Task variable found in Experiment 1 (see Results section below) persists even when the possible effect of these conjunctions is removed, it would support the idea that the contents of memory do not matter for attentional deployment in RSVP. Conversely, if a differential effect for the color and identity tasks is found with these simplified stimuli, it would provide evidence against the aforementioned account.

#### Participants

Nineteen new students (7 female, 12 male) participated in the experiment. Recruitment and selection procedures were identical to those in Experiment 1. Mean age was 20.8 years (range 19–24 years). One female participant was excluded from analysis by the same criterion as was used in Experiment 1. The mean age of the remaining eighteen participants was 20.6 years, again with identical range.

#### Apparatus and procedure

Experiment 2 was identical to Experiment 1, with the exception that colored discs were introduced to replace the colored digits in the memory set in the color task. The discs were otherwise uniform in appearance and similar in size to the digits they replaced.

### Experiment 3

In Experiment 3, a test of repetition priming was implemented. Depending on the Prime condition, one of the items from the memory set could be repeated as T2. The logic of this design was that if the contents of memory matter for temporal attention, a priming effect of this repetition may be expected and alleviate the attentional blink (i.e., cause an interaction effect with Lag, not just a main effect). If this effect is indeed observed, it would provide additional evidence that the interaction between memory and attention is content-driven, and not due to accessory systems (executive control or attentional gating). Note that the nature of priming implemented here was specifically tied to the memory set and the second target, and was independent of and orthogonal to the identity of T1. There was furthermore no opportunity for response priming effects to occur, as the responses to T1 and T2 were of a different kind (cf., [25]).

#### Participants

Nineteen new students (9 female, 10 male) participated in this experiment. The recruitment procedures and requirements were identical to those used in Experiment 1. Mean age was 21.6 years (range 18–29 years). Two participants (1 female, 1 male) were excluded from analysis using the same criterion as before. The mean age of the remaining 17 participants was 21.7 years, with the same range.

#### Apparatus and procedure

Experiment 3 was mostly identical to Experiment 1, except for the following differences. The memory set size of 1 was replaced by set size 2. This was necessary so that an item from the memory set could always be repeated as T2, without being identical to T1, even in case T1 matched an item from the memory set. The items from the memory set as well as T1 were now always red, and T2 was now black. The Task variable was replaced by the Prime variable (present or absent), indicating whether an item from the memory set was repeated as T2. This was the case in half the trials, which were again randomly mixed. As before, T1 and T2 were never identical. Finally, the Lag 1 condition was removed to reduce session time. The total number of trials came to 512, with an additional 32 practice trials.

## Results

### Experiment 1

**Figure 2:**
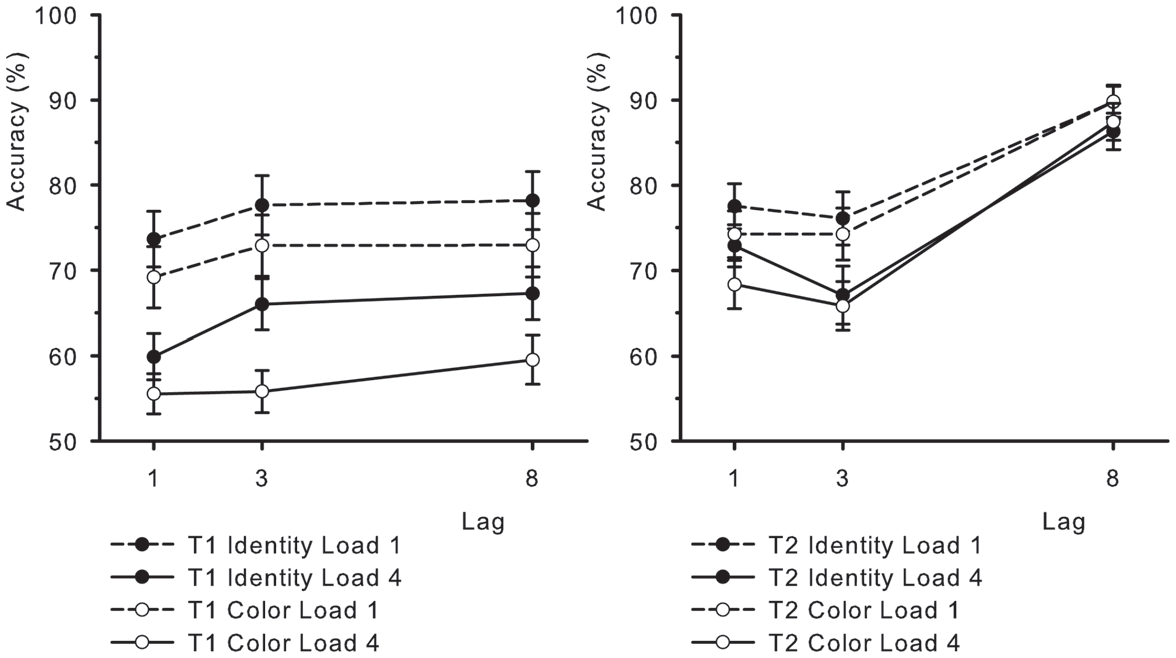
Identification accuracy in percent correct in Experiment 1. The left panel shows T1 performance, and the right panel shows T2 performance, given that T1 was correctly responded to (T2|T1), plotted as a function of T1-T2 Lag. Black symbols represent the identity task and white symbols represent the color task. Solid lines represent high memory load and dotted lines represent low memory load.

Performance on T1 was affected by Lag, *F*(2, 56) = 26.85, *MSE*  = .003, *p*<.001, Load, *F*(1, 28) = 58.9, *MSE*  = .027, *p*<.001, and Task *F*(1, 28)  = 5.91, *MSE*  = .055, *p*<.05. The lag effect on T1 showed classic competitive interference between T1 and T2 at Lag 1, where T1 performance dropped slightly to 64.6%, compared to 68.1% at Lag 3, and 69.5% at Lag 8 (cf., [26], [27]). Memory load also affected T1 in a predictable way; performance was better at low load (74.1%) than at high load (60.7%). Finally, the identity task was easier than the color task (70.4% vs. 64.3%). The interaction between Lag and Task was marginally significant, *F*(2, 56) = 2.6, *MSE*  = .003, *p*<.09. If anything, the interaction suggested that the difference between tasks was smaller at Lag 1 (4.4% difference in favor of the identity task) than at Lag 3 (7.4%) and Lag 8 (6.5%). None of the other interactions were reliable (*F*’s<2.2). The left panel of Figure 2 shows average performance on T1 as a function of Lag.

Performance on T2|T1 was similarly affected by Lag, *F*(2, 56)  = 48, *MSE*  = .022, *p*<.001, and Load, *F*(1, 28)  = 27.18, *MSE*  = .01, *p*<.001, but not by Task, *F*<1.8. The attentional blink was evident from the Lag effect, with performance reaching a low at Lag 3 (70.8%), compared to its peak at Lag 8 (88.3%). Lag 1 performance (73.2%) was slightly higher than it was at Lag 3, suggesting that a degree of Lag 1 sparing took place (see also [28]). The Load effect was straightforward; performance on trials with low memory load averaged 80.3%, compared to 74.6% on trials with high load. The interaction between Lag and Load was also reliable, *F*(2, 56)  = 5.26, *MSE*  = .005, *p*<.01. The interaction replicated the previously found result that memory load affects the attentional blink specifically [19]. The performance drop from low to high memory load at Lag 3 was 8.8%, compared to 5.3% at Lag 1, and 2.9% at Lag 8. The interaction between Lag and Task was again marginally significant, *F*(2, 56)  = 2.72, *MSE*  = .005, *p*<.08. The direction of this trend was that the differences due to Task were larger at Lag 1 (3.9% in favor of the identity task) than at Lag 3 (1.6%) or Lag 8 (−0.6%). None of the other interaction terms reached significance (*F*'s<1). The right panel of Figure 2 plots T2 performance as a function of Lag.

The data for the analysis of T2 performance were furthermore split between trials in which T1 matched the memory set and trials in which it did not. Though some numerical changes in the means were evident, this analysis did not change the pattern of results presented here. Overall, the results of the experiment were clear. First, the interaction between memory load and the attentional blink was replicated. Second, the color task was sufficiently difficult, so that performance on T1 was even lower than in the identity task, with a similar tendency showing for T2 performance. Despite the presence of these pre-requisite effects, there was no evidence for reliable differential effects of the memory task on attentional selection (i.e., the attentional blink was not affected by the Task variable). A possible explanation for these findings is that the interaction between the memory task and the attentional blink is due to a bottleneck at the level of control mechanisms, which may be required not only to deploy attention but also to access or update memory. In this scenario, the contents of working memory could be entirely inconsequential to the degree of interference that is observed between the tasks. This conclusion is as of yet premature, however, as there is another possible explanation for the present findings. In the current implementation of the color task, the items that were to be memorized were always conjunctions, that is, they contained both color and alphanumeric information. As is known from the Stroop interference effect [29], it is very difficult to ignore the alphanumeric property and focus on the stimulus color alone. Therefore, the possibility exists that no difference was found because the observers encoded and accessed the memory items by their conjunctions, rather than by the currently task-relevant property (i.e., color or identity) only. In order to examine this possibility, Experiment 2 was designed to remove the conjunctive nature of the memory items.

### Experiment 2

**Figure 3:**
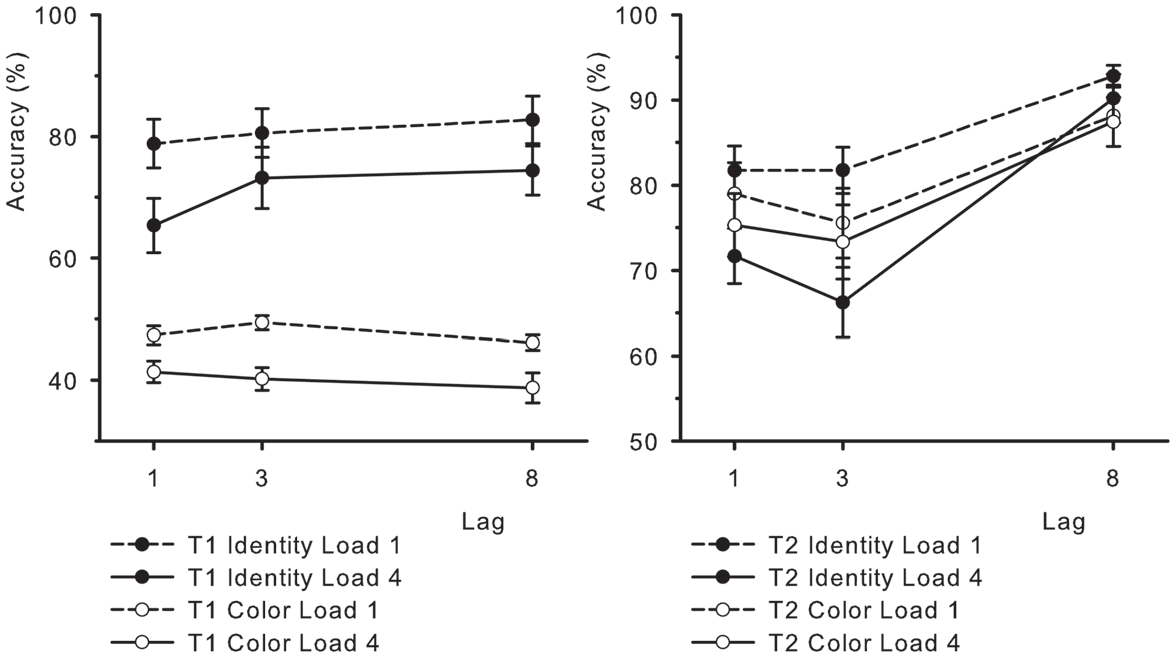
Identification accuracy in percent correct in Experiment 2. The left panel shows T1 performance, and the right panel shows T2 performance, given that T1 was correctly responded to (T2|T1), plotted as a function of T1-T2 Lag. Figure conventions are identical to those of Figure 2.

As before, there were significant effects of Lag, *F*(2, 34)  = 6.5, *MSE*  = .002, *p*<.005, Load, *F*(1, 17)  = 58.32, *MSE*  = .007, *p*<.001, and Task *F*(1, 17)  = 55.99, *MSE*  = .099, *p*<.001, on T1. T1 performance was slightly lower at Lag 1 (58.2%), compared to Lag 3 (60.8%) and Lag 8 (60.5%), replicating Experiment 1. The load effect was also replicated with higher performance (64.2%) under low memory load, compared to high load (55.5%). Finally, the color task averaged substantially lower performance on T1 (43.9%) than the identity task (75.8%). With regards to this performance, it should be noted here that the response screens to T1 and T2 were both time-limited to 1500 ms, and participants were not explicitly encouraged to guess in case they were uncertain about the targets. Trials on which no response was logged were classified as incorrect. Thus, even though there were only two response options for T1 (match or no match), it was possible for performance to drop below 50% due to trials on which no response was given. As mentioned, on the critical measure of T2 performance, analyses were performed with the prerequisite that T1 was correct. The left panel of Figure 3 plots T1 performance as a function of Lag.

There were reliable main effects of Lag, *F*(2, 34)  = 31.44, *MSE*  = .016, *p*<.001, and Load, *F*(1, 17)  = 17.42, *MSE*  = .01, *p*<.001, on T2|T1 accuracy. At the same time, Task had no overall effect (*F*<1). Mean performance was 76.9% at Lag 1, 74.2% at Lag 3, and 89.6% at Lag 8, reflecting an attentional blink with a degree of Lag 1 sparing, which was similar to Experiment 1. Performance with low memory load averaged 83.2%, while high memory load resulted in 77.4%. As expected, Lag and Load also interacted, *F*(2, 34) = 6.66, *MSE*  = .004, *p*<.005. High load impaired performance mostly at the short lags (6.8% difference at Lag 1, and 8.8% at Lag 3), compared to Lag 8 (1.7% difference). There was no reliable interaction between Lag and Task (*F*<2.5). Critically, however, the interaction between Task and Load was significant, *F*(1, 17)  = 6.39, *MSE*  = .011, *p*<.05. The interaction reflected a clear differential effect; memory load had a strong effect in the identity task, lowering performance by 9.4%, while in the color task this effect was only 2.2% in size. This was the case even though average performance in both tasks was very similar (80.7% in the identity task, and 79.8% in the color task). Finally, the three-way interaction between Lag, Load, and Task was also reliable, *F*(2, 34)  = 4.56, *MSE*  = .003, *p*<.05. This effect indicated that the differences due to the Task by Load interaction were restricted to the short lags. At Lag 8, the Load effect was comparable between the identity and color tasks (2.6% and 0.7% difference, respectively). At Lag 1 this was not the case, with a difference of 10% in the identity task, and 4.7% in the color task. Even more so, at Lag 3, the difference was 15.5% in the identity task, compared to 2.2% in the color task. When tested individually, neither of the differences in the color task were reliable (*t*<1.6). The right panel of Figure 3 plots T2 performance as a function of Lag.

The results of Experiment 2 were straightforward: When participants were accessing color information from memory, it did not increase the magnitude of the attentional blink, whereas retrieving alphanumeric information did increase blink magnitude. The effect was such, that even though the color task was noticeably more difficult (as can be seen from the means on T1), the blink was more pronounced in the identity task, that is, performance dropped below that of the color task. Thus, it would seem that the content of working memory does indeed interact with attentional deployment. Accessing information that is part of a different memory subsystem (i.e., visual memory vs. alphanumeric memory) does not impair attentional performance, while accessing the same type of information does (even without stimulus repetition).

These results are incompatible with the idea that generic executive control mechanisms or attentional gating functions are the bottleneck underlying the interaction between working memory and the attentional blink. Yet, there may be another possibility to salvage these accounts. One might assume that executive control functions or attentional gates could themselves be subdivided between different item categories or memory systems. Thus, although one control function may be used to process alphanumeric stimuli, another may be responsible for visual (color) information. Similarly, it could be assumed that searching for a digit amidst letter distractors is ‘dangerously’ similar to searching for a matching digit in a memory set, so that a careful attentional switch is needed, which might increase interference. The present data do not argue against these explanations. In order to fully establish a link between the contents of working memory and attention, it is necessary to establish the existence of content-driven effects in a situation that does not involve switching between different types of attentional gate. Although the existence of content-driven effects within a single attentional set would not per se disqualify the multiple-gate account as an account for the results so far, it would certainly become quite unparsimonious at the least.

### Experiment 3

**Figure 4:**
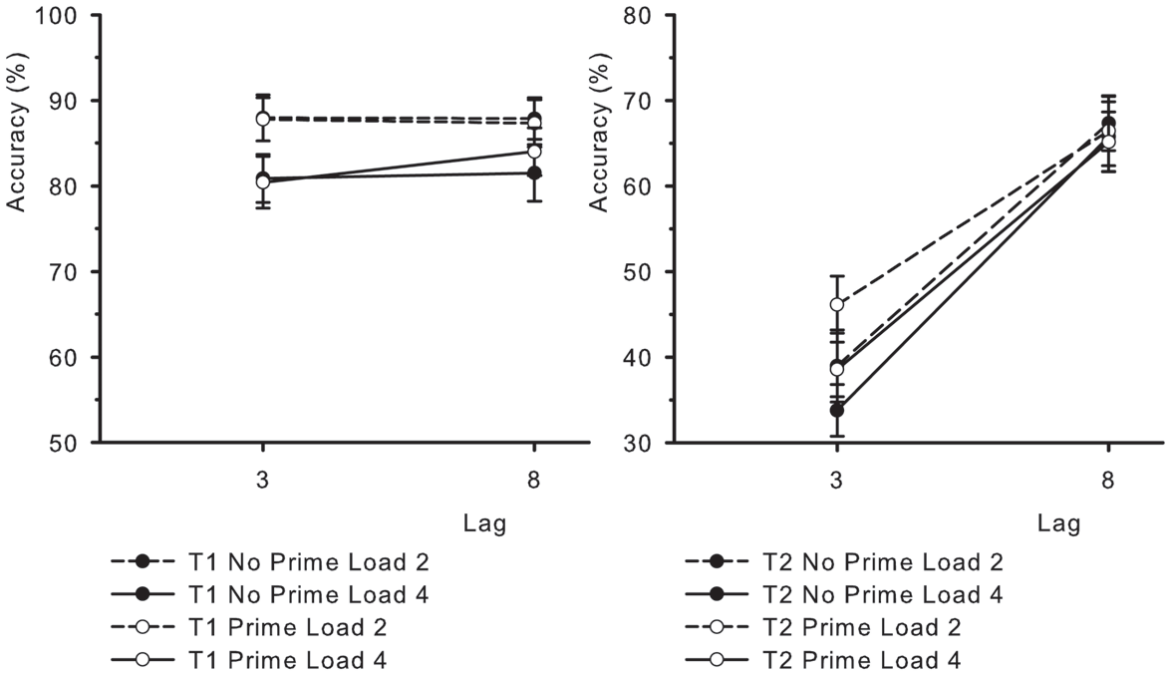
Identification accuracy in percent correct in Experiment 3. The left panel shows T1 performance, and the right panel shows T2 performance, given that T1 was correctly responded to (T2|T1), plotted as a function of T1-T2 Lag. Black symbols represent the condition without priming and white symbols represent the condition with priming. The remaining figure conventions are identical to those of Figure 2.

Load was the only variable to have a main effect on T1 performance, *F*(1, 16)  = 33.23, *MSE*  = .004, *p*<.001. Neither Lag nor Load was reliable (*F*'s<2.1). The interaction between Lag and Load was marginally significant, *F*(1, 16)  = 4.14, *MSE*  = .001, *p*<.06. It seemed to indicate that the Load effect might have been stronger at Lag 3 (7.2% difference) than at Lag 8 (4.8%). None of the other interaction terms were significant (*F*'s<2.1). The left panel of Figure 4 shows performance on T1 as a function of Lag.

Identification accuracy on T2 was reliably affected by Lag, *F*(1, 16)  = 59.52, *MSE*  = .041, *p*<.001, as well as by Load, *F*(1, 16)  = 8.46, *MSE*  = .006, *p*<.01. The Lag effect showed a sizeable attentional blink, with performance dropping to 39.4% at Lag 3, from 66.2% at Lag 8. The Load effect was due to lower performance with high memory load (50.8%) compared to low load (54.7%). The main effect of Prime was only marginally reliable, *F*(1, 16)  = 3.16, *MSE*  = .007, *p*<.09, but the trend was in the expected direction, with higher performance with priming (54.1%) than without (51.5%). The interaction between Load and Lag was again replicated, *F*(1, 16)  = 5.51, *MSE*  = .004, *p*<.05. Memory load had a larger effect at the short lag (6.4% difference) than at the long lag (1.5%). Critically, the interaction between Lag and Prime was highly significant, *F*(1, 16)  = 20.69, *MSE*  = .002, *p*<.001. The priming effect was clearly present at Lag 3 (5.9% benefit), but not at Lag 8 (−0.8%). The remaining interactions were not significant (*F*'s<1). The right panel of Figure 4 plots T2 performance as a function of Lag.

Finally, to investigate the possibility that the participants might have used a guessing strategy, we performed an additional analysis. Chris Olivers pointed out that observers could turn to selecting an item from the memory set in case they missed T2 altogether, since the prime condition was relatively frequent. In some conditions, this might indeed have increased task performance. To test the possibility that this could account for the priming effect, the Load 4 condition was selected for a comparison between guessing ‘normally’ and guessing from the memory set. Due to the distribution of T1-match and T2-match trials, guessing rates would be approximately equal here (at just above 14%). An ANOVA of this data still showed significant priming at Lag 3, *F*(1, 16)  = 5.66, *MSE*  = .003, *p*<.05, with a magnitude of 4.8%. In addition, an examination of the frequency of report of memory items in trials in which this was inappropriate (i.e., without T2 priming) showed that these were not prevalent (just over 20%, where chance would be just over 14%). Thus, this alternate guessing strategy cannot account for the priming effect.

The outcome of Experiment 3 thus confirmed that the contents of working memory can indeed affect attentional deployment. The repetition of an item that had been part of the previously memorized set facilitated the successful identification of T2. The absence of the priming effect at Lag 8, despite comparatively modest performance at that lag, and the presence of it at Lag 3 suggested that it might even be specific to the attentional blink. These results might appear to contradict those of Koelewijn, van der Burg, Bronkhorst, and Theeuwes [30], who showed a single prime item before an RSVP and observed lower performance for primes that matched T2, which they attributed to an instance of negative priming. There is critical difference between this study and the present one, however. In the present study, the memory items were accessed actively, and this was required at the time of T1, whereas in Koelewijn et al., the prime was held in memory throughout the RSVP, which likely created a degree of generic dual-task interference (see also [16], [17]).

## Discussion

The presently reported experiments showed how the contents of working memory have a specific influence on attentional deployment. First, the type of information in memory, and the categorical similarity to the stimuli of the attentional task, determined the degree of interference between working memory load and the attentional blink. Specifically, categorical overlap between working memory and attentional task increased blink magnitude, compared to an otherwise identical condition without categorical similarity. Second, when an item from the memory set was repeated as the second target in the attentional task, it attenuated the attentional blink.

The results are thus largely incompatible with theories that presume the locus of interference between working memory and attention lies at the level of executive control or attentional gating [8], [9]. If an executive control function was indeed necessary to access memory, there seems to be little reason to begin with why it would be different for specific types of information (in this case color and alphanumerical items). Indeed, the working memory model by Baddeley [24] features a universal “central executive”. However, the results of Experiment 1 and 2 could in principle be accounted for by a model that assumes different gates or control functions for different types of items. The interaction between these gates might explain the interactions presently observed, regardless of overall performance levels for each type of information. More problematic for such an account were the results of Experiment 3, which provided a clear indication that the contents of memory guided and in this case facilitated attentional selection even when only one type of information was used. An account that rests on the assumption that a single control function might underlie the interference observed between working memory load and attentional performance, as well as accounts resting on potential attentional set or gate switching effects, will be hard pressed to offer a viable explanation for the present priming effect. As noted, one might assume that searching for a digit amongst letter distractors is similar to matching a specific digit to a memorized set of digits, and that this similarity may complicate the attentional gate switching process, which could account for the results of Experiment 2 with some goodwill (although the lack of a main effect of task remains somewhat problematic). However, this account still fails to provide a reason for the priming effect observed in Experiment 3.

Taken together, theories of temporal attention that reject the idea that working memory is a causal factor in the attentional blink cannot easily explain the present results. The findings do support the principles of limited-processing (capacity-based) theories of the blink [4]–[6]. It should be noted that although the present results implicate working memory involvement in the attentional blink, this is not necessarily the same as memory consolidation. Of course, consolidation in memory certainly seems to be an instance of an active memory operation, but the present paradigm did not specifically implicate that particular function (i.e., consolidation was not differentially needed to do the memory task). Thus, theories that assume another type of causal mechanism for the interaction between working memory and attention (e.g., an inhibitory link; [4]) are able to accommodate the present results as well.

Finally, it seems likely that memory-related factors are not the sole cause of the blink, it is certain that other aspects of perception, such as visual masking [14], and ones that are task-or response-related [31], [32], contribute to the phenomenon. In this light, it would seem an interesting avenue for future research to quantify the relative contribution of each the underlying variables to the attentional blink.
